# A comparative evaluation of ChatGPT-assisted and traditional methods of teaching diagnostic assessments to medical students: A randomized controlled study

**DOI:** 10.1097/MD.0000000000050512

**Published:** 2026-09-04

**Authors:** Hua Xu, Yu Sun, Zhi-Wen Mo, Yu-Nan Man, San-Mao Liu, Yu-Ning Wei, Mao-Lin He

**Affiliations:** a Center for Education Evaluation & Faculty, Guangxi Medical University, Nanning, Guangxi Zhuang Autonomous Region, PR China; b Division of Spinal Surgery, The First Affiliated Hospital of Guangxi Medical University, Nanning, Guangxi Zhuang Autonomous Region, PR China; c School of Humanities and Management, Guangxi Medical University, Nanning, Guangxi Zhuang Autonomous Region, PR China.

**Keywords:** ChatGPT, diagnostic, medical education, Mini-Clinical Evaluation Exercise

## Abstract

The rapid development of artificial intelligence (AI) technology has brought new opportunities and challenges to the ever-evolving field of medical education. However, the specific impact of AI on the teaching of diagnostic assessments to medical students requires further investigation. The purpose of this study was to evaluate the effectiveness of ChatGPT-assisted teaching of diagnoses. Eighty medical students were randomly divided into 2 groups; 1 group received ChatGPT-assisted training, and the other group received conventional instruction. We evaluated the effectiveness of both teaching methods using a theoretical examination and the Mini-Clinical Evaluation Exercise (Mini-CEX). We also assessed satisfaction with the ChatGPT-assisted teaching method using a structured survey. The ChatGPT-assisted students scored significantly higher on the theoretical knowledge examination compared to the traditional teaching group (*t* = 6.34, *P* < .001). The ChatGPT-assisted group showed significant improvement on the Mini-CEX examination, particularly on medical history taking (Χ^2^ = 5.115, *P* = .024), doctor-patient communication (Χ^2^ = 5.051, *P* = .025), professionalism (Χ^2^ = 4.073, *P* = .044), clinical judgment (Χ^2^ = 4.503, *P* = .034), and overall capabilities (Χ^2^ = 7.218, *P* = .007). The majority of participants rated the ChatGPT teaching method highly and provided positive feedback, highlighting the benefits of interactive learning and skill acquisition. ChatGPT-assisted teaching not only improved the students’ scores on the theoretical knowledge of diagnostic assessments, but also enhanced the clinical knowledge of the medical students. The positive feedback of the ChatGPT-assisted group shows that this method has the potential to supplement and enhance conventional teaching methods in medical education. These results support further expansion and the application of AI in medical education.

## 1. Introduction

The continuous development of artificial intelligence (AI) is profoundly changing people’s lives and work habits, as it is integrated with education to offer unique advantages.^[1-3]^ As a natural language processing technology, ChatGPT automatically processes and generates natural language. Since its emergence, this technology has received much attention and it has been hailed for its epoch-making significance. ChatGPT also has prospects for broad application in the realm of medical education.^[4,5]^

The teaching of diagnoses serves as a bridge connecting basic medicine with clinical medicine, which is crucial for the cultivation of clinical skills and diagnostic reasoning skills of medical students.^[6]^ Traditional teaching methods mainly use a teacher-centered, 1-way approach. The learning process of the traditional teaching method is often dull and tedious and fails to engage students’ interests or develop their motivation, thereby leading to a decreased efficiency in knowledge acquisition and overall teaching effectiveness.^[7-9]^ This situation has resulted in numerous issues, such as a disconnect between teaching and practice, severely affecting medical students’ subsequent patient interviews and physical examinations, thereby failing to achieve the desired teaching outcomes.^[10,11]^ AI tools, such as ChatGPT, offer a promising solution because they can simulate complex patient interactions, thereby enhancing medical students’ interviewing, communication, clinical reasoning, and decision-making skills in various doctor-patient scenarios.^[12]^

ChatGPT is a generative AI that learns and simulates human conversational patterns, generating text that adheres to grammatical and semantic rules, and providing a new paradigm for medical training.^[13-15]^ Its proficiency in delivering instant, personalized feedback could fundamentally change the way teaching diagnostic assessments to medical students is conducted. Despite the recognized potential of AI in academia, there is limited empirical evidence that demonstrates its effects on the development of teaching diagnostic assessments to medical students. To fill this gap, this study aimed to explore the feasibility of applying ChatGPT to teaching diagnostic assessments and to investigate a new model for teaching diagnostic assessments to medical students.

## 2. Materials and methods

### 2.1. Study design

This study used a randomized controlled experimental design with blinded assessments. Specifically, the assessors were blinded to group assignments. They were unaware of whether students were in the ChatGPT-assisted group or the traditional teaching group during all stages of data collection and analysis. All post-course assessments were designed before the study began and were conducted consistently across groups. Assessors were sent anonymized student data to avoid group-label bias. Group assignments were managed by a third-party researcher who was not involved in data collection or assessment. A priori power analysis was conducted using G*Power 3.1. Based on a previous study investigating clinical examination scores (expected mean difference = 5.0, SD = 6.0), we calculated the required sample size for adequate statistical power. The results showed that a sample size of 36 participants per group would provide 80% power (α = 0.05, 2-tailed) for detecting a statistically significant difference using an independent t-test. To account for potential attrition (e.g., dropouts or missing data), we increased the sample size by 10%, which resulted in a final target of 40 participants per group. Subjects were randomly assigned to either the ChatGPT-assisted group (40 students) or the traditional teaching group (40 students). No significant differences were found in subjects’ gender (*P* = .32; 95% CI, −0.8 to 2.0), age (*P* = .65; 95% CI, −8.3% to 16.3%), or baseline clinical examination scores (*P* = .58; 95% CI, −1.6 to 2.8). All subjects had an equal opportunity to access the same teaching materials, instructors, and course intensity. The course textbook was the 9th edition of “Diagnostics,” published by the People’s Health Publishing House. Ethical approval (KY20250004) was obtained from the Ethics Committee of Guangxi Medical University, and informed consent was secured.

### 2.2. Subjects

This study assessed the effect of ChatGPT-assisted teaching on the theoretical and clinical skills of 80 undergraduate students from Guangxi Medical University in 2024. The cohort consisted of 32 males and 48 females who were randomly assigned using a computer-generated list.

### 2.3. Teaching groups

The instruction time was the same for both the traditional and ChatGPT-assisted groups.

#### 2.3.1. Preparation of the traditional teaching group

Instructors prepared typical cases for diagnostic assessments and included updated diagnostic knowledge. Multimedia presentations were developed to explain the diagnostic criteria for each disease.

The teaching process was divided into three stages:

1. Disease introduction and demonstration: The instructor initially introduced the disease, explaining the diagnostic process and emphasizing key aspects, such as history taking, physical examination, and laboratory tests.
2. Students independently conducted interviews, physical examinations, and laboratory tests with the instructor’s guidance.
3. At the conclusion of every session, the instructors offered tailored feedback regarding student performance and addressed students’ inquiries, thereby promoting an engaging and interactive educational atmosphere.

#### 2.3.2. Preparation of the ChatGPT-assisted teaching group

In addition to the traditional teaching content, the setup of ChatGPT (version 4.0) was involved. All the instructors received training in AI-assisted teaching before the teaching process.

The teaching process was divided into 4 stages:

1. Students became familiar with the features and possible diagnostic uses of ChatGPT 4.0, using it to explore dynamically generated clinical cases for diagnostic assessments. The AI effectively demonstrated communication strategies between the interviewer and patient in text form, enhancing the interactive experience of the interview.
2. Instructors reviewed students’ learning content and provided feedback to ensure the appropriate development of their diagnostic reasoning skills.
3. Students engaged in the independent practice of history taking, physical assessments, and laboratory tests with oversight from the instructor.
4. Instructors evaluated the student’s performance and responded to student’s inquiries in order to enhance their performance.

### 2.4. Instruments

#### 2.4.1. Evaluation of theoretical knowledge

At the end of the different teaching methods, the students were prepared for participation in the assessment of their theoretical knowledge. The academic affairs office prepared the examination questions for the assessment, using the theoretical exam format, with a highest possible score of 100 points. Strict surveillance of the students during the examination was conducted to prevent cheating.

#### 2.4.2. Evaluation of clinical knowledge

Mini-CEX is a repetitive and multipoint-oriented assessment that combines teaching and learning. It was proposed and is promoted by the American Board of Internal Medicine (ABIM) as a 2-way clinical assessment tool.^[16-18]^ This method involves systematizing and tabularizing information gained from assessment of the key areas of focus, multiple short-term evaluations of a single item, and combining the results of multiple evaluations to obtain the final assessment. The assessment is not entirely restricted to a specific location. At the selected time point, the designated assessor can directly observe the resident physician’s medical behavior, inquire about their actual medical practice, and then formulate the relevant treatment plan and implementation plan. The score can then be assigned through a preset table of the focus items, and feedback can be provided to the resident trainees in a timely manner. Because Mini-CEX provides more stable and reliable assessment of the skills and abilities of resident physicians than traditional assessment methods, it is now widely used in clinical teaching and assessment. All the raters were experienced clinicians with ≥5 years of teaching experience. Prior to data collection, the raters completed a standardized training session that included a detailed review of the Mini-CEX scoring rubric (9-domain scale, 1–9 points), with a focus on performance criteria for medical history, physical examination, doctor-patient communication, professionalism, clinical judgment, organizational effectiveness, and overall capabilities. The practice rating was based on simulated patient encounters (video-recorded cases) followed by group discussions to align interpretations. The raters also did a calibration exercise in which they independently scored 3 identical cases. Differences of >1 point were resolved through consensus to ensure uniform application of the scale. To assess reliability, 20% of the Mini-CEX assessments (n = 16) were randomly selected for dual rating by 2 independent raters who were blinded to each other’s scores. Continuous scores (overall performance) were analyzed using the intraclass correlation coefficient (ICC). The ICC for overall scores was 0.87 (95% CI, 0.81–0.92, *P* < .001), indicating excellent agreement between raters. After different training sessions, both groups of students participated in the Mini-CEX examination. Each indicator (medical history, physical examination, doctor-patient communication, professionalism, clinical judgment, organizational effectiveness, and overall capabilities) was scored on a 9-point scale, with higher scores indicating better student learning outcomes. A score of 7 or above was considered excellent, 4 to 6 was passing, and below 4 was failing.

1. Medical history taking: the student’s method of communication used by students to guide patients to report their medical history, the order of the guidance, the student’s response to various emotions and body language of the patients, and confirmation of information with the patients.
2. Physical examination: the selection of the physical examination items, the order of the physical examination, the method of examination, and the student’s response to the patient’s reactions.
3. Doctor-patient communication: analysis and explanation of the patient’s condition, explanation of any follow-up examinations or treatments, answers to patients’ questions, disease-related health education, and verbal and nonverbal eye and body communication.
4. Professionalism: subjects were assessed for their demonstrations of respect, compassion, empathy, building trust, concern for the patient’s comfort, maintaining confidentiality, adherence to ethical standards, understanding of legal aspects, and awareness of their professional limits.
5. Clinical judgment: combined with disease-related information, the differential diagnosis, preliminary diagnosis, and corresponding analysis of the patient, and formulation of a diagnosis and treatment plan.
6. Organizational effectiveness: the integrity, rationality, and order of the diagnosis, treatment processes, efficiency of clinical care, the ability to manage time, and report disease.
7. Overall capabilities: comprehensive assessment of the clinical competence of the residents throughout the observation process.

### 2.5. Satisfaction survey

After completing all training content, satisfaction of the participants in the ChatGPT group was surveyed about the training model and its effects. Instructors gathered the students and distributed questionnaires, which were completed and collected on the spot. All surveys on satisfaction administered to participants in the ChatGPT group were conducted anonymously. To ensure anonymity, no personal identifiers, such as names and student IDs, or any other information that could potentially reveal the identity of the respondents were collected or recorded on the questionnaires. The questionnaires were designed with only relevant questions related to the training model and its effects, and students were assured of their anonymity prior to filling out the questionnaires.

Student’s interviews: The interview responses were transcribed and analyzed using thematic analysis, a well-established qualitative research method. Two independent researchers first read through the transcripts to familiarize themselves with the data. Random sampling of both study groups was conducted for the semi-structured interviews, focusing on students’ benefits from the current teaching methods, overall ability and improvements, and their opinions and suggestions about the teaching. A 5-point rating scale (very satisfied, somewhat satisfied, dissatisfied, very dissatisfied, and uncertain) was used to evaluate the survey results, with satisfaction calculated as (the number of the very satisfied + the number of the somewhat satisfied)/ total number × 100%.

### 2.6. Statistical analyses

All data related to the observation indicators were collected, and SPSS 22.0 (IBM) statistical software was used to process the data. The results were classified, and different statistical tests were analyzed. Frequency count data are expressed as n (%), with between-group differences analyzed using the Chi^2^ test. Measurement data are expressed as (*x* ± *s*) with analysis of the between-group differences using the *t* test and *P* < .05 indicating statistical significance. In addition, we applied Bonferroni correction to adjust the significance level for multiple comparisons. The adjusted α level was set to 0.05.

## 3. Results

### 3.1. Theoretical knowledge exam scores of the 2 groups of medical students

After the different teaching sessions, both groups of medical students underwent theoretical knowledge examinations, with the ChatGPT-assisted group achieving an average score of 85.38 ± 2.88 points, which was significantly higher than the average score of the traditional teaching group at 80.30 ± 4.15 points (*t* = 6.34, *P* < .001). These results indicate that the ChatGPT-assisted group benefited from learning diagnostic theory.

### 3.2. Comparison of the Mini-CEX scores between the 2 groups of medical students

The average time for all trainees to complete the Mini-CEX examination was 37.92 ± 1.03 minutes, with an average of 6.00 ± 0.55 minutes for immediate feedback. Compared to the traditional teaching group, the ChatGPT-assisted group showed significant improvements in medical history taking, doctor-patient communication, professionalism, clinical judgment, and overall capabilities. Detailed comparisons of the Mini-CEX scores between the 2 groups are shown in Table 1.

**Table 1:** The scale scores of Mini-CEX evaluation between the ChatGPT-assisted group and the traditional teaching group.

Item	Traditional teaching group, cases (%)	ChatGPT‑assisted group, cases (%)	*P*-value
Total cases	40	40	
Medical history taking			.024
Meets expectation	22	12	
Exceeds expectation	18	28	
Physical examination			.823
Meets expectation	19	18	
Exceeds expectation	21	22	
Doctor-patient communication			.025
Meets expectation	27	17	
Exceeds expectation	13	23	
Professionalism			.044
Meets expectation	26	17	
Exceeds expectation	14	23	
Clinical judgment			
Below expectation	2	1	.034
Meets expectation	22	16	
Exceeds expectation	16	23	
Organizational effectiveness			.822
Meets expectation	23	22	
Exceeds expectation	17	18	
Overall capabilities			
Meets expectation	27	15	.007
Exceeds expectation	13	25	

ChatGPT = Chat Generative Pre-Trained Transformer, Mini-CEX = Mini-Clinical Evaluation Exercise.

### 3.3. Satisfaction survey results of the ChatGPT-assisted group

The satisfaction survey revealed that participants were very satisfied with and interested in the ChatGPT-assisted teaching method; there were no reports of dissatisfaction. Table 2 summarizes these survey results, including the specific aspects of the teaching method that the students rated highly.

**Table 2:** Subject satisfaction assessment of the ChatGPT-assisted teaching.

Items	Very satisfied (n)	Satisfied (n)	Neutral (n)	Dissatisfied (n)	Satisfaction (%)
Improving learning efficiency	38	2	0	0	100
Stimulating an interest	35	4	1	0	97.5
Improving communication skills	36	2	2	0	95
Deeper understanding of clinical skills and theoretical knowledge	38	0	2	0	95
Enhancing students self-learning abilities	37	3	0	0	100
Helping to achieve learning objectives	34	5	1	0	97.5

ChatGPT = Chat Generative Pre-Trained Transformer.

## 4. Discussion

Applying ChatGPT to the diagnostic teaching of medical students is a key step in the enhancement of their learning and their future educational activities and projects using AI. This study found that AI-assisted teaching played an important role in diagnostic assessments. The better performance of the ChatGPT-assisted group indicates that AI can serve as a supplementary educational tool. The ChatGPT-assisted group demonstrated significant improvements in their Mini-CEX examinations. These abilities are essential for the comprehensive development of clinical practitioners, suggesting that interactive learning environments really do help in learning how to diagnose patients. Participants expressed satisfaction with the ChatGPT-assisted learning approach, confirming that AI-assisted teaching enhanced student’s engagement. This positive feedback may stem from the effective personalization and interactivity of ChatGPT and its ability to cater to the diverse learning styles of medical students.^[19,20]^ However, a heavy reliance on AI and the balance of interactivity is also worth considering. The advantages of the AI-assisted method were demonstrated in students’ performance on the Mini-CEX examinations, indicating the reproducibility and adaptability of the teaching scenarios provided by ChatGPT. Additionally, ChatGPT can offer targeted answers, suggestions, and relevant knowledge points based on medical students’ feedback and questions, thereby promoting improved learning outcomes.^[21-23]^ This study explored the application of AI in the teaching and assessments of the diagnostic clinical abilities of the different groups of students using the Mini-CEX.

However, the single-center setting of this study reduces the generalizability of the conclusions. Moreover, the development of AI and medical education requires continuous exploration of the potential applications of ChatGPT in medical teaching.^[24,25]^ Future research should evaluate the role of ChatGPT in medical education using large samples, multi-centers, and longitudinal studies. However, some limitations of ChatGPT-assisted teaching may hinder its application. Firstly, regarding overreliance on AI, we have added a section discussing how students may become overly dependent on ChatGPT for tasks such as assignment completion and knowledge acquisition, potentially hindering the development of critical thinking, independent research, and problem-solving skills. This overdependence could also lead to a lack of active engagement with course materials and a diminished ability to learn from failures. Secondly, we have explored the issue of misinformation. ChatGPT, despite its advanced language generation capabilities, can produce incorrect or misleading information. We speculate that this might have a negative impact on students, especially when they may not have the expertise to verify the accuracy of the information provided by the AI tool. Additionally, we have considered the potential for ChatGPT to reinforce existing biases in its responses, which could have negative implications for the diverse student population in terms of equality in education. Finally, issues regarding data protection, student’s rights, risk balance, ethical orientation, and values should also be addressed to ensure the sustainable application of AI in medical education.

The application of ChatGPT in medical students’ diagnostic assessments is clearly feasible, allowing students to simulate real diagnostic scenarios, access rich teaching resources, enhance diagnostic skills, and accumulate practical experiences. However, there are limitations to its use, such as the inability to replace real medical scenarios and issues related to accuracy and reliability. Therefore, when using ChatGPT for teaching diagnostic assessments to medical students, we need to consider how and when to use it in teaching, along with appropriate measures to address its shortcomings to improve teaching effectiveness.

## 5. Conclusions

Overall, AI-assisted teaching contributes to the advancement of medical students’ theoretical and practical skills. The extremely high satisfaction of the study’s participants indicates that ChatGPT-assisted teaching is feasible and has positive implications for improving medical students’ learning outcomes.

## Author contributions

**Data curation:** Yu Sun.

**Formal analysis:** Yu Sun.

**Project administration:** Yu-Nan Man.

**Software:** Zhi-Wen Mo.

**Supervision:** Zhi-Wen Mo, Yu-Nan Man, San-Mao Liu, Yu-Ning Wei.

**Validation:** San-Mao Liu.

**Visualization:** Yu-Ning Wei.

**Writing – review & editing:** Mao-Lin He.

**Writing – original draft:** Hua Xu.
